# Formulation and Development of an Experimental Polishing Paste with Antimicrobial Activity Based on *Coturnix coturnix* (Codorniz) Eggshell

**DOI:** 10.1155/2021/9998989

**Published:** 2021-07-06

**Authors:** Frank Mayta-Tovalino, Alicia Fernández-Giusti, Joyce Del Pino, Daniel Alvitez-Temoche, Roman Mendoza, Abigail Temoche, Arnaldo Munive-Degregori

**Affiliations:** ^1^PhD Department of Health Sciences, Faculty of Medicine, Universidad Nacional Mayor de San Marcos, Lima, Peru; ^2^Postgraduate Department, Change Research Working Group, Faculty of Health Sciences, Universidad Científica del Sur, Lima, Peru; ^3^Natural Sciences Laboratory, Faculty of Health Sciences, Universidad Científica del Sur, Lima, Peru; ^4^Academic Department, Universidad Nacional Federico Villarreal, Lima, Peru; ^5^Academic Department of Rehabilitative Stomatology, Faculty of Dentistry, Universidad Nacional Mayor de San Marcos, Lima, Peru

## Abstract

**Aim:**

To formulate and develop a new experimental polishing paste based on *Coturnix coturnix* eggshell and to evaluate its abrasive, remineralizing, and antibacterial activities.

**Materials and Methods:**

The research was experimental, longitudinal, comparative, and prospective. To measure the antibacterial efficacy, analysis units consisted of wells were made. The microorganisms *S. aureus, E. coli, E. faecalis, C. albicans*, and *S. mutans* were inoculated with experimental paste (*Coturnix coturnix* quail eggshell base) and control paste (Universal Polishing and Diamond Excel) consisting of *n* = 12 for each group.

**Results:**

It was found that, among the strains inoculated, the quail paste presented with the highest antimicrobial effectiveness to *C. albicans* and *S. mutans* with an average of 8.70 ± 0.14 and 11.65 ± 0.15 mm, respectively. On the other hand, the Universal Polishing paste only had an average of 7.00 ± 0.11 and 8.71 ± 0.11 mm for *C. albicans and S. mutans*, respectively. Significant differences were observed only in these two strains *p* < 0.001.

**Conclusions:**

The quail paste demonstrated antimicrobial efficacy against *C. albicans* and *S. mutans* compared to control paste, Diamond Excel, and Universal Polishing according to the time and type of microorganism.

## 1. Introduction

Currently, tons of eggshells are discarded everyday causing environmental problems such as unpleasant odor and flies and other vectors of communicable diseases. Different mechanisms are being used worldwide to recycle eggshell wastes. Typically, eggshell wastes go to a landfill, fertilizer, feed additive, adsorbent, calcium supplement, paper making etc. Therefore, these mechanisms can be more sustainable techniques to manage the waste by potential use in dental science [1–3].

Eggshells are traditionally used in different industrial processes for agricultural engineering with the main objective of correcting the pH of some acidic soils. Currently, this method has an economic value. Similarly, mollusk shells can also be an alternative source of calcium carbonate (CaCO_3_) to reduce the depletion of natural limestone reserves, which are irreplaceable [4, 5]. Despite the mentioned utility of this product, eggshells have yet to gain enough attention regarding recycling into materials in health sciences. In this context, it is essential to investigate the potential use of the mineral protein from eggshells [5].

On the other hand, eggshell powder has been studied recently. Its potential use as a source of calcium (Ca) has been demonstrated since it contains approximately 39% elemental CaCo_3_. This eggshell powder could promote cell differentiation and increase bone mineral density, giving its potential in the treatment and prevention of certain bone diseases [6–8].

Another important point to consider is the roughness of the surface of the restorative materials because it is a primary parameter that can induce the accumulation of a biofilm that could subsequently negatively affect oral health [9, 10]. Evidence indicates that smooth and polished prosthetic restoration surfaces are more successful intraorally [11].

Thus, this study aimed to develop and formulate a novel experimental polishing dentifrice based on *Coturnix coturnix* eggshell and to evaluate its antimicrobial efficacy.

## 2. Materials and Methods

This research would be sent to the Ethics Committee of the Universidad Científica del Sur for review and for authorization (no: 174-CIEI-AB-CIENTÍFICA-2020). No ethical conflicts are anticipated because this is a purely experimental in vitro study.

### 2.1. Sample Size

The sample size was calculated based on the means comparison formula. The STATA^®^ 15.0 statistical software was used. For this sample calculation, a beta of 0.8 and an alpha of 0.05 were used. A total of 60 wells, divided among the 5 experimental groups, were considered, with *n* = 12 wells for each group.

  Group A: *S. aureus* ATCC® 25923 ™ compared to the 4 pastes (control paste, quail paste, Universal Polishing paste, and Diamond Excel paste)
  Group B: *E. coli* ATCC® 25922 ™ compared to the 4 pastes (control paste, quail paste, Universal Polishing paste, and Diamond Excel paste)
  Group C: *E. faecalis* ATCC® 29212 ™ compared to the 4 pastes (control paste, quail paste, Universal Polishing paste, and Diamond Excel paste)
  Group D: *C. albicans* ATCC® 10231 ™ compared to the 4 pastes (control paste, quail paste, Universal Polishing paste, and Diamond Excel paste)
  Group E: *S. mutans* ATCC® 25175 ™ compared to the 4 pastes (control paste, quail paste, Universal Polishing paste, and Diamond Excel paste)

### 2.2. Preparation of Eggshell Powder

The eggshells were collected from a poultry farm in Lima, Peru. They were then washed and disinfected for six hours in a diluted sodium hypochlorite solution. The eggshells were vacuum-dried at 37°C for 24 hours and were ground with a mill until a fine-textured powder was obtained. Sodium lauryl surfactant was mixed to the eggshell powder (4.5 kg) to improve its solubility in water. The eggshell powder was then placed in a 500 ml flask and was dry-ground at 400 rpm for forty minutes until about 0.3 mm fine grains were obtained.

### 2.3. Dentifrice Preparation

**Figure 1:**
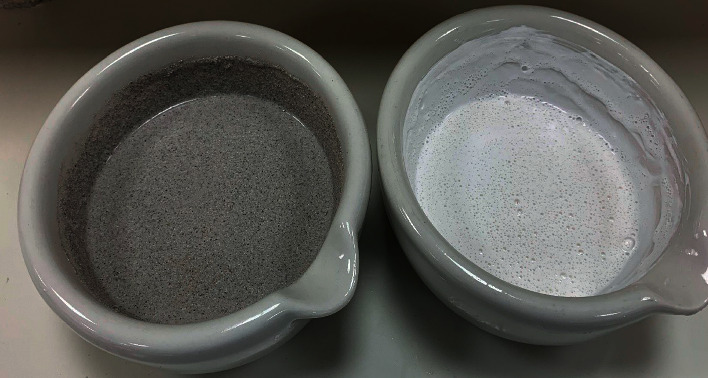
Preparation of experimental quail toothpaste and control toothpaste.

The quail eggshell was crushed until reaching 125 g. The other components described were weighed according to the proportions in Table 1 and added to a mortar (tetrasodium pyrophosphate, Aerosil, Nipagin, saccharin, menthol, and titanium dioxide). All the components contained in the mortar were mixed until a homogeneous mixture was obtained to incorporate the Goma and fluoruro solutions. When the mixture obtained a good viscosity, glycerin was then incorporated, and finally, distilled water was incorporated as well. Finally, all the components were mixed until a good paste viscosity was achieved. The whole process was completed by labeling the product in an amber-colored glass container (Figure 1).

### 2.4. Antimicrobial Efficacy

**Figure 2:**
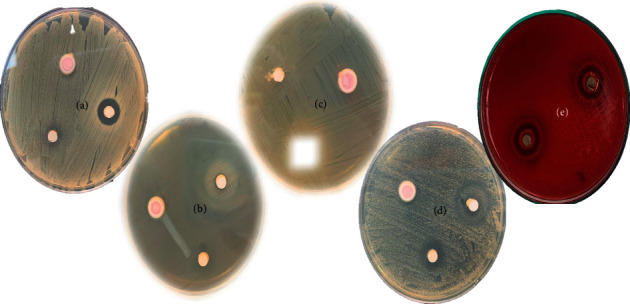
Measurement of inhibition halos against the different microorganisms evaluated. Group A: *S. aureus* ATCC® 25923™; group B: *E. coli* ATCC® 25922™; group C: *E. faecalis* ATCC® 29212™; group D: *C. albicans* ATCC® 10231™; and group E: *S. mutans* ATCC® 25175™.

The microbial strains were obtained from GenLab and were cultured on Mueller–Hinton agar (Difco Laboratories, USA). Only the *S. mutans* agar had 5% defibrinated sheep blood at 42°C in 10% CO_2_. The microorganisms were stored in the MH medium with 20% glycerol. The minimum inhibitory concentration (MIC) was determined using the broth microdilution method. A control medium was prepared; it was compared to a bacterial suspension approximation of 0.5 McFarland CFU/ml. Finally, the inhibition halos were measured by Kirby–Bauer method (Figure 2).

### 2.5. Statistical Analyses

For the elaboration of the descriptive analysis, the measures of central tendency and dispersion of the numeric variables will be obtained. In addition, normality will be determined using the Shapiro–Wilk test. Depending on this, for the bivariate analysis, Mann–Whitney *U*-test or Student's *t*-test will be used. The statistical analysis will be carried out in the STATA 15 program, establishing a level of significance of *p* < 0.05.

## 3. Results

It was found that the quail paste presented antimicrobial effectiveness against S*. aureus, E. coli, E. faecalis, C. albicans*, and *S. mutans* strains with an average of 12.63 ± 0.08; 12.68 ± 0.07; 12.71 ± 0.07; 8.70 ± 0.14; and 11.65 ± 0.15 mm, respectively. On the other hand, the Universal Polishing paste only had an average of 7.00 ± 0.11 and 8.71 ± 0.11 mm for *C. albicans and S. mutans*, respectively. Significant differences were seen in these two strains only (*p* < 0.001) (Table 2).

## 4. Discussion

Calcium carbonate (CaCO_3_) from eggshells could provide substitute minerals used in paper treatment to improve its surface brightness, opacity, and strength. This chemical compound can also be used to enhance the texture and appearance of the paper among other functions such as abrasives on different textures [9, 12, 13].

Although there are currently some toothpaste formulations that have great antimicrobial activity [14], there is always a growing social demand to generate new and natural compounds for the care and maintenance of oral health [15]. For example, there are certain natural herbal toothpastes that contain sodium bicarbonate and various components that claim to have medicinal properties such as chamomile extract, which has anti-inflammatory and antimicrobial properties; echinacea extract, which increases immune response; mentha piperita extract, which has antiseptic properties; and sage extract, which decreases tissue bleeding [16, 17]. Therefore, this research aimed to demonstrate the effectiveness of eggshell in the field of stomatology.

Although there is a diverse use of eggshell, there is limited evidence available on its potential as an abrasive powder on denture polishing. In addition, calcium could play an important role in the process of enamel remineralization and has very high bioavailable calcium [8, 18]. Hence, calcium carbonate is mainly used as a base for different magisterial medicinal preparations [8, 19, 20].

Regarding antimicrobial activity, the present study found that the quail eggshell toothpaste had optimal antimicrobial activity against multiple oral cavity strains such as *C. albicans, S. mutans,* S*. aureus, E. coli,* and *E. faecalis*. Similarly, the study by Verkaik et al. [21] discussed the antibacterial efficacy of different toothpastes with natural antimicrobial components and compared it with chlorhexidine. The antibacterial efficacy was evaluated against *S. oralis* and *A. naeslundii*, which are two important colonizers of the surface enamel. They concluded that herbal toothpastes have immediate and continuous antibacterial properties like chlorhexidine gluconate. Another study by Smolarek et al. [22] also stated that toothpastes with natural compounds have clinical therapeutic potential. Our study showed consistent results with the previous studies; these various commercially available types of toothpaste of natural origin have different antimicrobial activities.

Guven et al. [23] investigated six different kinds of toothpaste and a newly formulated single-brand mouthwash and five kinds of toothpaste and three commercially available mouthwash to determine their antimicrobial activity against some oral microorganisms, such as *C. albicans* and *S. mutans*. Although it was found that both formulations showed antimicrobial activity for some microbes, more studies are required.

This study has some limitations. Scientific literature [24, 25] on toothpaste based on quail eggshell was scarce, which hinders a deeper interpretation of the results. Another limitation was the need for specialized culture media to cultivate some facultative anaerobic microorganisms. However, we were able to replicate the microbiological assays. Finally, a large amount of quail eggshell was needed due to the small size of the shell.

## 5. Conclusions

Statistically significant differences were only found in antimicrobial efficacy between quail paste and Diamond Excel, Universal Polishing, and control paste against *C. albicans* and *S. mutans.*

## Figures and Tables

**Table 1:** Formulation and development of the experimental polishing dentifrice.

Components	Quantity	×5	Properties
Eggshell-powdered quail	25.0 g	125 g	Abrasive
Tetrasodium pyrophosphate	3.75 g	18.75 g	Additives
Aerosil (hydrophilic fumed silica)	0.20 g	1.00 g	Additives
Nipagin (sodium methylparaben)	0.15 g	0.75 g	Preservative
Crystallized sodium saccharin	0.20 g	1.00 g	Sweetener
Menthol crystals	0.80 g	4.00 g	Flavoring
Titanium dioxide (dye)	0.53 g	2.65 g	Binder
Xanthan gum	1.05 g	5.25 g	Whiteness to toothpastes
Sodium lauryl ether sulfate (LESS), 28%	2.00 g	10.00 g	Surfactants
Sodium fluoride (fluoride)	0.32 g	1.60 g	1450 ppm fluorine
Liquid glycerine	24.00 mL	120.00 mL	Moisturizer
Distilled water	42.00 mL	210.00 mL	Moisturizer
Total	100.00	500.00	

**Table 2:** Comparison of the antimicrobial efficacy of quail paste versus others according to time and type of microorganism.

Group	Microorganism	Time				*p* ^*∗*^	*p*
		24 hours		48 hours			
		Mean	SD	Mean	SD		
Control paste	*S. aureus*	12.53	0.16	12.33	0.13	0.619	>0.05^‡^
Quail paste		12.63	0.08	12.33	0.16	0.433	
Universal Polishing paste		0	0	0	0	—	
Diamond Excel paste		0	0	0	0	—	

Control paste	*E. coli*	12.61	0.08	12.56	0.10	0.740	>0.05^‡^
Quail paste		12.68	0.07	12.36	0.10	0.678	
Universal Polishing paste		0	0	0	0	—	
Diamond Excel paste		0	0	0	0	—	

Control paste	*E. faecalis*	12.61	0.75	12.28	0.11	0.945	>0.05^‡^
Quail paste		12.71	0.07	12.31	0.14	0.841	
Universal Polishing paste		0	0	0	0	—	
Diamond Excel paste		0	0	0	0	—	

Control paste	*C. albicans*	6.53	0.30	6.16	0.16	0.840	<0.001^†^
Quail paste		8.70	0.14	8.43	0.12	0.924	
Universal Polishing paste		7.00	0.11	7.00	0.11	—	
Diamond Excel paste		11.10	5.44	13.41	0.27	0.051	

Control paste	*S. mutans*	11.45	0.31	10.71	0.43	0.835	<0.001^†^
Quail paste		11.65	0.15	11.21	0.29	0.160	
Universal Polishing paste		8.71	0.11	8.25	0.18	0.841	
Diamond Excel paste		11.25	1.17	10.3	0.49	0.001	

^*∗*^Shapiro–Wilk test. ^⁑^Student's *t*-test (groups with null values were excluded from the analysis). ^†^ANOVA test. All measurements were expressed in mm, using the Kirby–Bauer method.

## Data Availability

The data of this research will be available with prior authorization of the corresponding author.
